# Retinal Origins of Aberrant Salience in Schizophrenia: The Perceptual Theory

**DOI:** 10.1016/j.bpsgos.2026.100764

**Published:** 2026-06-04

**Authors:** Petr Adámek, Veronika Rudolfová, Kristýna Malenínská, Jiří Horáček

**Affiliations:** a PeThrs Lab, Center for Advanced Studies of Brain and Consciousness, National Institute of Mental Health, Klecany, Czechia; b Third Faculty of Medicine, Charles University, Prague, Czechia; c Center for Quantitative Health, Center for Genomic Medicine and Department of Psychiatry, Massachusetts General Hospital, Boston, Massachusetts; d Department of Psychiatry, Harvard Medical School, Boston, Massachusetts; e Laboratory of the Neurophysiology of the Memory, Institute of Physiology of the Czech Academy of Sciences, Prague, Czechia

**Keywords:** Dopamine, Neurodevelopmental, Retina, Salience, Schizophrenia, Visual perception

## Abstract

In schizophrenia research, visual processing abnormalities have traditionally been viewed as downstream consequences of cortical dysfunction. However, the epidemiological observation that congenital cortical blindness appears to confer protection against schizophrenia, while peripheral blindness, regardless of its developmental timing, does not, indicates that visual input itself may play a more fundamental role in disease pathogenesis. Here we propose the Perceptual Theory of Schizophrenia (PerTh), a framework positing that instability in retinal dopamine signaling acts as an initiating mechanism in a subset of individuals. Retinal dopamine functions as a master gain controller, modulating receptive field properties and spatial frequency processing through D_1_ and D_2_ receptor–mediated mechanisms. We propose that schizophrenia-associated genetic liabilities, encompassing dopaminergic, GABAergic (gamma-aminobutyric acidergic), neuroimmune, and other risk factors, converge on retinal dopamine homeostasis, producing chronically unstable signals that corrupt the sensory foundation upon which predictive models of reality are built. This noisy input cascades through thalamocortical pathways, disrupting physical and cognitive salience formation; driving compensatory synaptic sprouting; and ultimately rendering diffuse, weak connections vulnerable to complement-mediated hyperpruning during adolescence. The framework integrates retinal electrophysiology, Bayesian predictive processing, and neurodevelopmental pruning mechanisms to explain symptom emergence timing, the predominance of auditory over visual hallucinations, and phenotypic heterogeneity. PerTh does not seek to replace existing dopaminergic, glutamatergic, neuroimmune, or neurodevelopmental models but instead complements them by identifying a potential upstream contribution originating in the visual periphery. This perspective generates testable predictions and points to novel research directions targeting retinal function in psychosis.

## Plain Language Summary

In this article, we propose a new theory about how schizophrenia develops, focusing on the retina’s role in visual processing. It suggests that unstable dopamine levels in the retina create noisy visual signals, which disrupt how the brain processes important information. These disruptions may lead to psychotic symptoms, such as delusions and hallucinations. The theory offers a fresh perspective on schizophrenia and highlights the need for research into retinal dysfunctions as a possible early contributor to the disorder’s development.

Abnormalities of visual perception have increasingly been recognized in schizophrenia research. Early work emphasized magnocellular pathway impairments and low-level visual integration; more recent findings have extended these abnormalities to the retina itself, including altered contrast sensitivity, visual acuity, and electrophysiological responses (1, 2, 3). Such anomalies are traditionally seen as downstream consequences of cortical dysfunction rather than as contributors to disease etiology.

This view began to shift following epidemiological observations concerning congenital blindness. In a population-based cohort of >467,000 individuals, no schizophrenia cases were reported among 66 individuals with congenital cortical blindness, which is visual loss from bilateral occipital lesions while ocular structures remain intact. No comparable protection was observed for peripheral blindness arising from ocular dysfunction (4, 5, 6). These findings are statistically underpowered, given the rare comorbidity, and do not constitute definitive evidence. Nevertheless, the consistent absence of reported cases raises questions that existing models do not explicitly address.

This distinction between cortical and peripheral blindness is mechanistically significant for our framework. In congenital cortical blindness, retinal output remains intact at the eye but cannot reach the cortical areas where salience is computed and predictive models are formed. No retinal signal, whether noisy or clean, propagates through thalamocortical pathways to drive aberrant salience formation. In contrast, peripheral blindness arising from ocular dysfunction often preserves partial or qualitatively degraded retinal activity (7). According to the Perceptual Theory of Schizophrenia (PerTh), it is precisely such degraded but preserved retinal signaling that drives the pathogenic cascade. Causes of complete peripheral congenital blindness (e.g., bilateral anophthalmia) are too rare to permit epidemiological testing of whether the complete absence of retinal output, regardless of anatomical origin, would confer similar protection.

The relationship between visual function and psychosis risk is complex. Large longitudinal studies have yielded contradictory findings: In a Swedish cohort of >1 million men, it was found that poorer uncorrected visual acuity increased psychosis risk, while corrected refractive errors in a comparably sized Israeli cohort were associated with reduced schizophrenia risk (8,9). The discrepancy likely reflects whether corrected or uncorrected acuity was assessed, indicating that the critical variable is signal quality and stability rather than visual impairment per se. Recent Mendelian randomization analyses further indicate that schizophrenia may itself be a causal risk factor for poorer visual acuity (10). Such bidirectional patterns are consistent with retinal dopaminergic signaling acting as a shared mechanism, given its established role in regulating both contrast sensitivity and visual acuity.

Several questions emerge from these observations. Why do many individuals with schizophrenia exhibit visual processing deficits that appear to predate psychosis onset (11)? Why does the disorder typically manifest during adolescence, coinciding with critical periods of visual system maturation (12)? In addition, why do antipsychotics that target cortical and subcortical dopamine receptors (13) also affect retinal contrast sensitivity (14, 15, 16, 17)? The literature shows some heterogeneity. Unmedicated first-episode patients tend to show elevated contrast sensitivity (18,19), whereas chronic medicated patients show reduced sensitivity that scales with antipsychotic dose (20,21). This pattern indicates that retinal dopamine plays a direct role in the pharmacological action of antipsychotics and that medication status is a critical confounder when interpreting contrast sensitivity findings in schizophrenia.

**Figure 1:**
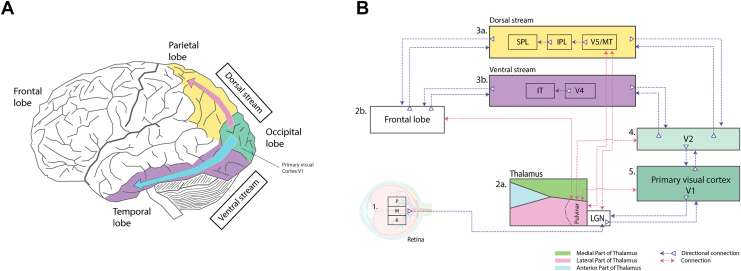
The visual pathway and proposed sequence of aberrant signal propagation in the PerTh framework. **(A)** Anatomical overview of the 2 major visual processing streams. The dorsal stream (yellow) projects from the occipital lobe through the parietal cortex and processes spatial location and movement. The ventral stream (purple) projects from the occipital lobe through the temporal cortex and processes object identity and form. **(B)** Circuit organization and proposed PerTh cascade. Retinal ganglion cells project through P, M, and K channels to the LGN of the thalamus and onward to V1, V2, and the dorsal and ventral streams. The thalamic pulvinar provides additional connectivity between cortical visual areas and the frontal lobe. Numbered annotations indicate the proposed sequence of pathophysiological changes: 1) retinal dopamine instability generates an aberrant, noisy signal; 2a/2b) the thalamus and frontal lobe are among the first regions to show structural change as they fail to adapt to unstable input; 3a/3b) changes subsequently spread into the dorsal and ventral streams; 4, 5) V2 and V1 show later and typically smaller effects, consistent with their earlier developmental maturation and strong continuous sensory drive that preserves synaptic architecture. IPL, inferior parietal lobule; IT, inferior temporal cortex; LGN, lateral geniculate nucleus; P/M/K, parvocellular/magnocellular/koniocellular channels; PerTh, perceptual theory of schizophrenia; SPL, superior parietal lobule; V1, primary visual cortex; V2, secondary visual cortex; V4, visual area 4; V5/MT, middle temporal area.

We propose PerTh, according to which in at least a subset of individuals, genetically driven instability in retinal dopamine signaling generates persistently noisy visual input that cascades through thalamocortical pathways, thereby disrupting salience formation and contributing to excessive synaptic pruning during adolescence (22). A schematic overview of the visual pathway and the proposed sequence of signal propagation is provided in Figure 1.

This retina-first hypothesis does not seek to replace existing dopaminergic, glutamatergic, neuroimmune, or neurodevelopmental models. It complements them by highlighting a potential upstream contribution that has received relatively little attention. The framework generates falsifiable predictions and points to research directions targeting the visual periphery.

## Situating PerTh Within Existing Theoretical Frameworks

Each of the current models of schizophrenia pathophysiology contribute important insights. The dopaminergic hypothesis highlights subcortical hyperdopaminergia and the efficacy of D_2_ receptor antagonists (23,24). Glutamatergic models emphasize NMDA receptor hypofunction and disrupted cortical inhibition (25,26). Neurodevelopmental accounts integrate genetic vulnerabilities with environmental factors and adolescent synaptic pruning (27). Neuroimmune models implicate complement-mediated synaptic elimination, microglial overactivation, and proinflammatory signaling, with *C4A* (complement component 4A) structural variants representing one of the strongest individual genetic risk factors identified to date (28).

Large-scale genomic studies have broadened this landscape. Genome-wide association study (GWAS) meta-analyses implicate >280 risk loci across dopaminergic, glutamatergic, synaptic, calcium channel, and transcriptional regulation genes (29). Postmortem transcriptomic analyses have revealed dysregulation of synaptic, mitochondrial, and immune modules (30). Single-cell analyses have shown schizophrenia polygenic risk converging on retinal amacrine cells (31). Thus, individual dopaminergic variants explain only a small fraction of disease risk, and pathogenesis involves convergent disruption across multiple molecular pathways.

Existing models have not explicitly addressed peripheral sensory processing, particularly retinal function, in disease pathogenesis. PerTh identifies an upstream contribution that interfaces with established frameworks: Chronically noisy retinal signals could contribute to the excitatory-inhibitory imbalance emphasized in glutamatergic models or represent a peripheral manifestation of the same genetic risk variants implicated in central dopaminergic dysfunction. Therefore, PerTh is complementary and applicable particularly to individuals with prominent visual processing abnormalities.

We acknowledge that direct evidence causally linking each stage of the proposed cascade remains limited. Each cascade element, such as retinal dopamine dysfunction, visual processing deficits, aberrant salience, and excessive synaptic pruning, is independently supported empirically in schizophrenia, but their sequential causal relationships have not yet been established. Therefore, the framework represents mechanistically plausible but empirically unproven causal links requiring coordinated validation. We outline such a research program in Future Research Directions, with testable predictions probing each cascade stage independently and in sequence.

## The Retinal Dopamine Cascade: From Retinal Dysfunction to Psychosis

**Figure 2:**
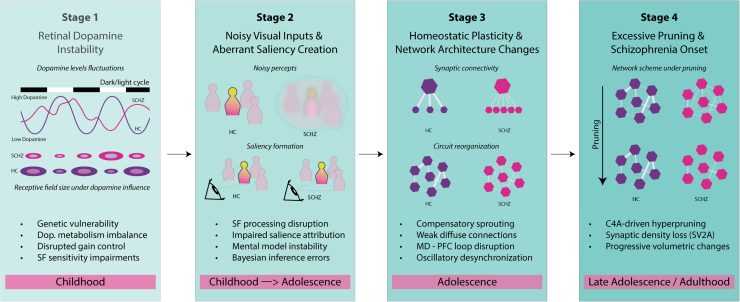
Retinal-origin cascade linking early visual dysfunction to adolescent synaptic pruning and schizophrenia onset. The figure summarizes the framework detailed in the article (see stages 1–4 in The Retinal Dopamine Cascade: From Retinal Dysfunction to Psychosis). *C4A*, complement component 4A; HC, healthy control; MD, mediodorsal thalamus; PFC, prefrontal cortex; SCHZ, schizophrenia; SF, spatial frequency; SV2A, synaptic vesicle glycoprotein 2A.

Current theories treat sensory abnormalities as downstream consequences of cortical or subcortical dysfunction. However, the cortical blindness paradox suggests that vision itself may be a primary driver of schizophrenia. This reframing, with sensory dysfunction at the beginning rather than the end of the pathological sequence, may explain why absence of visual processing is linked to reduced schizophrenia risk. We propose a 4-stage mechanistic cascade linking retinal dopamine dysfunction to schizophrenia symptomatology, grounded in molecular and cellular mechanisms (Figure 2).

## Stage 1: Retinal Dopamine Instability—A Proposed Initiating Mechanism

### Molecular Architecture of Retinal Dopamine

**Figure 3:**
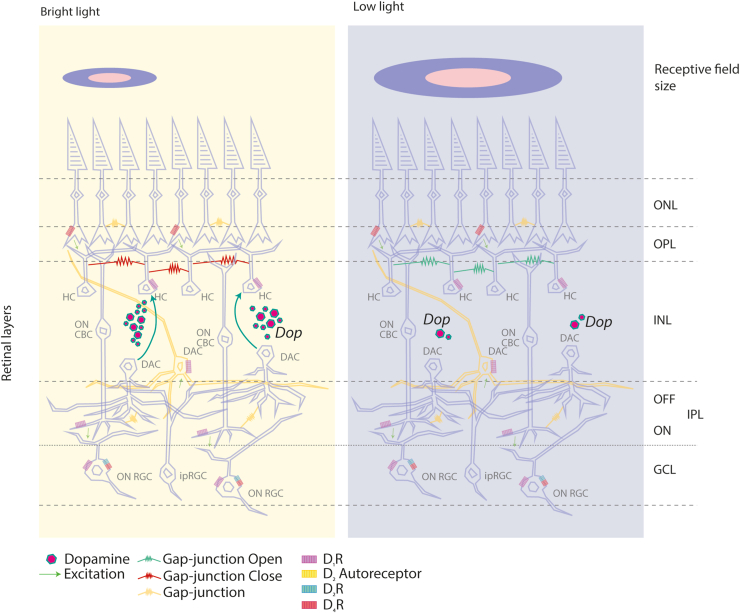
Retinal dopamine gain control across bright light and low light conditions. The figure summarizes the proposed role of dopamine as a master retinal gain controller that strengthens surrounds in bright conditions and relaxes them in darkness, thereby regulating spatial-frequency sampling from coarse to fine detail. Schematic of retinal circuitry from outer to inner layers (ONL, OPL, INL, IPL, GCL) with OFF/ON IPL sublaminae. Left: bright light (photopic). Right: low light (mesopic-scotopic). Cones drive ON CBCs via HCs, amacrine cells, and DACs. In bright light, higher dopamine tone (turquoise dots) from amacrine sources increases D_1_ receptor signaling on BCs, HCs, DACs, and GCs and strengthens receptive-field surrounds (push-pull gain). In low light, dopamine tone falls, D_1_–dependent surround antagonism weakens, and network coupling/gap-junction state shifts accordingly. The inset at right illustrates the corresponding change in receptive-field size. CBC, cone bipolar cell; DAC, dopaminergic amacrine cell; GCL, ganglion cell layer; HC, horizontal cell; INL, inner nuclear layer; IPL, inner plexiform layer; ipRGC, intrinsically photosensitive retinal ganglion cell; ONL, outer nuclear layer; OPL, outer plexiform layer.

The retina contains the highest peripheral dopamine concentration outside the central nervous system (32), synthesized by dopaminergic amacrine cells (DACs) located between the inner nuclear and plexiform layers (33). Although DACs constitute <1% of all retinal cells, they exert disproportionately broad influence through volume transmission. Dopamine released extrasynaptically reaches receptors across all retinal layers and virtually all neuronal classes (34,35). In the primate retina, DACs are absent from the foveola but reach peak density in a parafoveal ring approximately 2 to 3 mm from the fovea, with density declining toward the midperipheral retina (36,37). Combined with volume transmission, this distribution makes dopaminergic modulation most prominent in macular and parafoveal circuits and weaker peripherally. Retinal dopamine acts via D_1_ receptors on bipolar, horizontal, amacrine, and ganglion cells and via D_2_ receptors on photoreceptors and DACs themselves (34, 38) (Figure 3).

### The Gain Control Mechanism

Retinal dopamine functions as a master gain controller, adjusting visual sensitivity to ambient conditions and behavioral demands. In cone bipolar and ganglion cells, D_1_–mediated modulation of GABA_A_ (gamma-aminobutyric acid A) receptors via PKA (protein kinase A) phosphorylation adjusts receptive-field surround strength, strengthening surround antagonism under bright conditions and weakening it in darkness (39) (Figure 3). This produces push-pull spatial frequency tuning. D_2_ receptors govern fine-detail processing at moderate-to-high frequencies (∼2.3 cycles/degree), and D_1_ receptors govern low-frequency coarse-feature sampling (∼0.5 cycles/degree) (40).

### Genetic Vulnerability and Dopamine Instability

In genetically vulnerable individuals, schizophrenia-associated variants converge on retinal dopamine homeostasis. Classical dopaminergic variants such as *COMT* Val158Met (41) and *DRD2* polymorphisms (42) represent one mechanism, but the vulnerability extends well beyond dopaminergic genes (43,44). Individual common variants confer modest effects, with the polygenic risk score explaining approximately 7% of liability variance, with single nucleotide polymorphism–based heritability of 24% (29). These variants gain pathogenic relevance through convergent disruption of dopaminergic signaling, which we propose acts as a final common pathway integrating diverse genetic liabilities. GWAS-based cell-type enrichment analysis demonstrates that schizophrenia polygenic risk accumulates robustly in retinal amacrine cells, with associated genes including *DRD2*, *CACNA1I* (calcium voltage-gated channel), *DOC2A* (calcium-dependent neurotransmitter release), and *SLC32A1* (vesicular GABA/glycine uptake) (31). The enrichment was not specific to any amacrine subtype, indicating that genetic vulnerability spans GABAergic and broader synaptic mechanisms converging on disrupted dopamine homeostasis. Pattern electroretinography^a^ in healthy control participants demonstrates that even acute dopamine perturbations (e.g., D_2_ antagonism with sulpiride) selectively impair spatial frequency processing (17).

This genetic architecture helps explain why visual processing deficits in schizophrenia exceed what dopaminergic variants alone would predict. Several additional processes can independently disrupt retinal dopamine homeostasis. GABAergic dysregulation of amacrine networks destabilizes the timing and magnitude of DAC dopamine release. Neuroimmune activation involving microglial and complement-mediated mechanisms implicated in cortical pathology (28,45) affects retinal synaptic integrity. The retina’s high metabolic demand and light exposure render it susceptible to oxidative stress, which impairs dopamine synthesis and receptor function, while redox dysregulation has been proposed as a convergence hub for genetic and environmental risk (46). Epigenetic modifications linked to prenatal and early-life exposures further modulate dopaminergic and synaptic gene expression (47). These mechanisms likely operate in combination, with different pathways predominating across the heterogeneous schizophrenia population.

## Stage 2: Noisy Visual Inputs and Aberrant Salience Formation

Salience comprises 2 components. Physical salience describes how certain stimulus features automatically capture attention through bottom-up, stimulus-driven processes independent of current goals (48). Cognitive salience involves top-down, goal-directed attention allocation guided by task demands or internal objectives (49). Because cognitive salience builds on physical salience, disrupted sensory sensitivity impairs the ability to attribute appropriate meaning to environmental stimuli (50).

### Physical Salience Disruption

Unstable retinal dopamine creates inconsistent receptive field properties, generating noisy visual signals at cortical inputs. These fluctuations also affect spatial frequency processing. Low spatial frequencies (LSFs) carry coarse, global information about shape, orientation, and scene layout. High spatial frequencies (HSFs) carry fine-grained details such as edges, textures, and object features. LSF information is rapidly extracted (within 100–170 ms of stimulus onset) and transmitted to the prefrontal cortex, where it informs top-down predictions guiding attention, object recognition, and higher cognitive processes (51, 52, 53). Within the PerTh framework, unstable retinal gain control disrupts both LSF-based scene analysis and subsequent HSF-mediated refinement of percepts, impairing attentional capacity and the ability to rapidly incorporate salient percepts into the stream of consciousness (22).

Vision’s neuroanatomical dominance (54) makes it the primary sensory foundation for predictive models of reality. As the most peripheral component, the retina is the critical trigger point, and dysfunction here propagates through the entire visual hierarchy. In contrast, congenital deafness does not confer comparable protection against schizophrenia, with prevalence in prelingually deaf individuals matching the general population (55,56). The cortical versus peripheral distinction documented for blindness has no direct epidemiological counterpart for deafness, because central auditory disorders are anatomically rare and not separated from cochlear deafness in existing literature. The cochlea does receive dopaminergic input from the lateral olivocochlear system (57), but this neuromodulatory architecture is more circumscribed than retinal dopamine, which acts as a master gain controller across all major retinal cell types through volume transmission. The absence of a protective effect of deafness is thus consistent with this architectural difference and with vision’s substantially greater cortical representation.

### Cascade to Cognitive Salience

As described previously (22), physical salience disruption prevents accurate cognitive salience formation. In Kapur’s framework (58), dysregulated dopamine alters the threshold at which sensory events become salient. PerTh identifies retinal dopamine as the source, with fluctuating gain control causing inappropriate “pop out” of irrelevant stimuli while failing to highlight informative signals. Perceptual organization deficits cause processing of object parts rather than unified wholes, activating inappropriate semantic associations and potentially explaining the loosening of associations and thought disorder characteristic of schizophrenia (59). Fragmented visual percepts thus cascade into language dysfunction, providing a mechanistic link between retinal dopamine instability and verbal-conceptual symptoms of psychosis.

### Mental Model Instability

Inconsistent visual inputs corrupt formation of stable mental models. In the hierarchical Bayesian framework, the brain updates beliefs by combining sensory evidence with prior expectations, with dopamine modulating precision weighting (60). Retinal dopamine dysfunction introduces noise that prevents reliable signals from being distinguished from unreliable ones, forcing the developing brain to constantly revise priors based on corrupted data (22). Compensatory mechanisms paradoxically increase confidence in flawed models, treating uncertain inferences as highly precise predictions.

In the PerTh, Conrad’s delusional mood represents the moment when lifelong compensation fails under adolescent cognitive demands (61). Patients do not suddenly perceive novel abnormalities; they experience the conscious consequences of cumulative predictive failures. Because much early visual processing occurs outside awareness, high-risk individuals may not recognize that their experience differs from that of others (62). Retinal dysfunction, present since birth and perceptually normalized, cannot be identified as the source of mounting unease, and patients externalize the discrepancy as a diffuse change in the world. Crystallization into frank delusions during apophany represents a catastrophic attempt to resolve this uncertainty through explicit causal explanations (61,63).

## Stage 3: Homeostatic Plasticity and Synaptic Architecture Changes

### Compensatory Sprouting

Faced with inconsistent visual inputs, neurons cannot strengthen specific synaptic pathways through repeated activation. They compensate by forming numerous weak connections across many input sources, attempting to extract meaningful patterns from unreliable signals (64). Such incoherent input patterns can decouple strong synaptic connections and shift networks toward broadly distributed, low-strength connectivity (65), impairing signal-to-noise discrimination, reducing computational efficiency, and creating vulnerability to excessive pruning during adolescence.

Compensatory sprouting may appear to contradict the principle that biological systems minimize energy expenditure. However, we argue that sprouting driven by chronically noisy input is maladaptive rather than adaptive. Unlike homeostatic plasticity in intact circuits, which refines connectivity to maximize efficiency, the reorganization here generates diffuse, energetically costly circuits failing to achieve stable representations. Empirical evidence supports this. Unmedicated patients with active positive symptoms show elevated frontal cortical glucose metabolism (66), and hippocampal hypermetabolism appears in individuals at high risk for psychosis prior to onset (67). Long-term adaptation to noisy peripheral signals may eventually lead to neurotoxic processes and reduced pyramidal connectivity (1), with initial compensatory hypermetabolism giving way to the frontal hypometabolism and synaptic loss characteristic of chronic schizophrenia (68).

### Circuit-Level Reorganization

Compensatory sprouting alters cortical circuit architecture, particularly in prefrontal regions where mental models of reality are maintained (69,70), providing a mechanistic explanation for the connectivity abnormalities observed in schizophrenia (71, 72, 73). Postmortem studies and in vivo positron emission tomography (PET) using the SV2A (synaptic vesicle glycoprotein 2A) biomarker confirm widespread reductions in synaptic density, with pronounced effects in the prefrontal and anterior cingulate cortices (74, 75, 76). These changes are present even in antipsychotic-naïve early-onset patients, indicating that they precede treatment and chronic illness (77,78). We propose that these synaptic alterations stem from the cascade of unstable bottom-up signals, manifesting most severely in prefrontal areas required to integrate degraded sensory information into coherent representations of reality.

### Thalamocortical Disruption

Effects extend beyond the visual cortex through thalamocortical loops, particularly impacting the mediodorsal thalamus (MD) and its reciprocal connections with the prefrontal cortex. The lateral geniculate nucleus, receiving direct retinal input, must process increasingly noisy signals. Disruption propagates beyond visual pathways: The MD, an essential partner to the prefrontal cortex in cognition that amplifies cortical representations during demanding tasks, also receives degraded sensory information through corticothalamic feedback loops (79). This destabilizes not only visual processing but also the timing and coordination of thalamocortical oscillations critical for attention, working memory, and sensory gating. Computational models show that even modest increases in input noise can desynchronize thalamic relay neurons and corrupt cortical activation patterns (80, 81, 82).

## Stage 4: Excessive Pruning and Symptom Emergence

### The Adolescent Vulnerability Window

During adolescence, experience-dependent synaptic pruning refines cortical circuits by eliminating weak or unused connections while strengthening active ones (83,84). In healthy development, this is guided by consistent patterns of correlated activity reinforcing behaviorally relevant circuits (85). In individuals with retinal dopamine dysfunction, the cortex has already adapted to chronically noisy inputs through homeostatic mechanisms. Drawing on principles of structural homeostasis (64), we propose that persistent visual signal instability drives pyramidal neurons to form numerous but weaker contacts attempting to maintain stable activity (65). During adolescence, these diffusely connected, low-strength synapses are preferentially eliminated through activity-dependent pruning (85,86).

### Complement-Mediated Hyperpruning

Genetic vulnerabilities amplify this process through the complement system, which tags synapses for microglial elimination during development (45,87, 88, 89). Schizophrenia-associated *C4A* variants increasing *C4A* expression are associated with elevated disease risk (28,90). However, high *C4A* expression alone is insufficient, as many unaffected individuals carry high-expression variants, and the relationship between *C4A* levels and brain volume differs between patients and healthy control participants, indicating compensatory mechanisms in resilient individuals (91,92).

We propose that retinal dopamine dysfunction provides the critical second hit; chronically noisy visual inputs drive formation of diffuse, weak synaptic connections; and high *C4A* expression amplifies vulnerability only when combined with this weakened architecture. The SV2A PET evidence of synaptic loss in antipsychotic-naïve patients (stage 3) is consistent with early hyperpruning of compromised circuits.

### From Synaptic Loss to Symptom Emergence

Longitudinal neuroimaging in patients with schizophrenia reveals that gray matter loss follows a specific spatiotemporal pattern, beginning in thalamic hubs before cascading into frontal, temporal, and parietal cortices (93,94). Relative sparing of occipital and early visual cortex, despite a hypothesized retinal contribution, is consistent with the predominance of abnormalities in heteromodal temporofrontal cortices (95) and with the heterogeneous, often smaller, occipital effects reported (96). This pattern aligns with homeostatic plasticity principles; regions receiving strong, continuous sensory drive maintain synaptic strength via activity-dependent global synaptic scaling (97). Developmentally, occipital regions complete key pruning phases by early adolescence, whereas the prefrontal cortex remains plastic into late adolescence and the third decade (98,99). Therefore, dysfunction originating in visual pathways spreads through anatomically connected regions, with timing determined by each area’s developmental trajectory and pruning schedule (1).

As excessive pruning reduces synaptic density below critical thresholds, structural loss translates into psychotic symptoms. Subtle cognitive inefficiencies are detectable during childhood, before clinical diagnosis (100). During adolescence and early adulthood, accelerating aberrant pruning generates positive symptoms acutely through immediate perceptual misinterpretation and sustained cognitive elaboration into delusions, reflecting predictive machinery struggling to maintain coherence with unreliable inputs precisely when complement-mediated synaptic elimination peaks.

As illness progresses, negative symptoms (avolition, alogia, anhedonia, social withdrawal, and diminished emotional expression) become prominent. These arise from cumulative loss of cortical computational capacity through excessive pruning combined with disrupted local excitatory-inhibitory balance. Loss of GABAergic interneuron function and altered pyramidal connectivity impairs prefrontal-limbic circuits regulating motivation and emotional expression (101,102). In approximately half of patients, negative symptoms persist throughout the illness course, reflecting irreversible loss of synaptic infrastructure (103). Cognitive symptoms become apparent as adult demands exceed diminished capacity, reflecting convergence of original noisy signal processing, hyperpruning-driven synaptic loss, and disruption of thalamocortical oscillations essential for working memory and attention.

Within this framework, the relapsing-remitting course of schizophrenia reflects structural vulnerability established by excessive pruning. Once synaptic density falls below critical thresholds, cortical circuits lack computational reserve to maintain stable processing under environmental challenge. Well-documented relapse precipitants, including psychosocial stress, sleep deprivation, and substance use (104,105), can overwhelm this diminished system. Striatal dopamine sensitization further enhances reactivity during acute episodes (106). Within the PerTh framework, retinal dopamine instability continues to act as a persistent source of degraded sensory input, while postonset relapses are primarily determined by the interaction between residual cortical capacity and environmental demands.

## The Auditory Hallucination Paradox

If retinal dysfunction drives schizophrenia pathogenesis, why do patients predominantly report auditory rather than visual hallucinations? This paradox can be resolved by considering how sensory modalities undergo perceptual adaptation.

Visual disturbances developing gradually from childhood undergo perceptual normalization. The brain recalibrates baseline expectations to accommodate slowly deteriorating input, leaving patients unaware of lifelong deficits in contrast sensitivity or visual acuity (107). In contrast, auditory anomalies, particularly misattributed inner speech, emerge acutely and intermittently (108), resisting normalization and remaining consciously salient. Therefore, patients may report hearing voices while failing to recognize concurrent visual distortions that have been experienced since childhood.

Auditory hallucinations also typically arise from failures in self-monitoring of inner speech rather than peripheral sensory dysfunction (109,110). When forward model mechanisms fail to tag self-generated verbal thoughts as internal, these are experienced as external voices. Within the PerTh framework, retinal dopamine instability generates noisy visual signals that, through the cascade in stages 2 to 3, ultimately destabilize the self-monitoring circuits distinguishing internal from external signals. This mechanism is independent of retinal pathology, explaining why auditory symptoms predominate even when visual dysfunction initiates the pathogenic cascade. Evolutionary threat detection circuits may further amplify auditory anomalies, as ancestral survival depended on detecting auditory signals of predators or social threats (111, 112).

## Future Research Directions

The PerTh framework generates testable predictions across multiple experimental scales. Four complementary approaches probe distinct cascade links: 1) noninvasive screening combining smooth pursuit eye tracking with longitudinal pattern electroretinography to establish retinal dopamine biomarkers in adolescents at psychosis risk; 2) chemogenetic manipulation of retinal DACs in animals during adolescence, testing whether retinal dopamine instability produces schizophrenia-relevant phenotypes following established neurodevelopmental criteria; 3) induced pluripotent stem cell–derived retinal cells from patients with schizophrenia to distinguish intrinsic cellular pathology from upstream dopaminergic dysregulation; and 4) intervention studies targeting retinal dopamine stabilization, complement-mediated pruning, or synaptic plasticity at developmentally specific windows. Detailed protocols are provided in the Supplement.

## Limitations

Several limitations must be acknowledged. First, epidemiological evidence remains statistically inconclusive. Danish register data following 460 cases of early blindness across 2,500,332 individuals found fewer than 5 schizophrenia cases (113). Power calculations indicate that demonstrating complete protection would require approximately 3 million individuals, while detecting 50% risk reduction would require approximately 11 million. Larger international collaborations would be necessary for cortical blindness specifically.

Second, current data cannot exclude the possibility that retinal and central dopamine dysfunction occur in parallel rather than sequentially. Schizophrenia-associated variants affecting dopamine metabolism could simultaneously alter retinal and brain dopaminergic signaling, making retinal changes a biomarker rather than a causal driver. If the absence of schizophrenia in congenital cortical blindness is confirmed in larger studies, this would indicate that visual input itself, not parallel dopaminergic dysfunction, is necessary for disease expression in at least some individuals.

Third, schizophrenia’s heterogeneity challenges any unifying model. Visual symptoms occur in approximately 60% of cases (114), ranging from 4% to 80.3% across studies depending on assessment and onset (115,116). Large-scale GWASs implicate glutamatergic genes (e.g., *GRIN2A*) at least as strongly as dopaminergic ones (29,117). Within the PerTh framework, glutamatergic dysfunction represents one of several upstream contributors converging on retinal dopamine homeostasis (stage 1). The framework is most directly applicable to individuals with prominent retinal dopamine instability, irrespective of which upstream genetic pathway predominates.

Fourth, complement-mediated hyperpruning is not unique to schizophrenia. Excessive synaptic elimination has been implicated in bipolar disorder with psychotic features, and *C4A* expression correlates with delusion severity across psychotic disorders (118). We propose that the schizophrenia phenotype emerges from combined excessive pruning and lifelong accumulation of aberrant salience signals from retinal dopamine instability, distinguishing it from bipolar disorder, in which hyperpruning may interact with affective circuit dysregulation to produce episodic rather than chronic psychosis.

Finally, the mechanism underlying symptom variability remains unclear. Retinal dysfunction may cause simple visual phenomena, while complex hallucinations require additional cortical disruption (119). Outcomes ranging from minor distortions to frank psychosis probably reflect interactions between retinal dysfunction and modifying factors, including polygenic risk burden, cognitive reserve (120), developmental stress exposure (121), and endophenotypic variation in sensory processing (122).

## Conclusions

The PerTh framework offers a perspective on schizophrenia pathogenesis in which sensory processing abnormalities, specifically retinal dopamine instability, contribute to disease development in a subset of individuals rather than merely representing downstream consequences of cortical dysfunction. While epidemiological observations regarding congenital cortical blindness remain statistically underpowered, they raise questions warranting further investigation.

Whether the PerTh represents a schizophrenia-specific mechanism or a transdiagnostic vulnerability for psychosis is a key issue to explore. Retinal abnormalities clearly extend beyond schizophrenia. Optical coherence tomography meta-analyses have documented retinal nerve fiber layer and ganglion cell layer thinning in both schizophrenia and bipolar disorder (123,124). Electroretinogram profiles overlap across these disorders but show partially distinct patterns (125). Psychophysical deficits including impaired contrast sensitivity surround suppression (126), visual backward masking (127), and contour integration (128) correlate with psychosis severity independently of diagnosis. Notably, visual backward-masking deficits are present in schizoaffective and bipolar disorder but not in depression or abstinent alcoholics (129), indicating that these impairments track psychosis liability rather than psychiatric illness in general. However, recent GWAS-based cell-type enrichment analysis shows schizophrenia polygenic risk converging robustly on retinal amacrine cells, whereas bipolar disorder shows no consistent retinal cell-type association (31). Therefore, the genetic architecture appears to be at least partially diagnosis specific. We propose PerTh as primarily applicable to the schizophrenia spectrum, while retinal dopamine instability may represent a broader predisposing factor whose clinical expression is shaped by additional genetic, epigenetic, and environmental modulators. Longitudinal retinal biomarker studies spanning the psychosis spectrum represent a priority for testing this hypothesis.

Ultimately, the value of PerTh lies not in claiming to explain all cases of schizophrenia but rather in stimulating new research directions examining peripheral sensory processing in psychosis development. By directing attention to retinal function as a potential contributor to, rather than merely a marker of, disease pathophysiology, we hope to open new avenues for understanding the origins of this devastating disorder.

## Footnotes

a Noninvasive technique measuring retinal ganglion cell function.
