# Supplementary material for: Retinal Origins of Aberrant Salience in Schizophrenia: The Perceptual Theory
Source: Biol Psychiatry Glob Open Sci. 2026 Jun 4;6(5):100764. doi: 10.1016/j.bpsgos.2026.100764 (PMC13380484; doi:10.1016/j.bpsgos.2026.100764)
Supplement: Supplemental Text [file mmc1.pdf]

SUPPLEMENTARY INFORMATION

Retinal Origins of Aberrant Salience in Schizophrenia: The Perceptual Theory

Adámek *et al.*

Supplementary Section S1: Expanded Future Research Directions

The PerTh framework generates testable predictions and identifies multiple intervention points across the developmental cascade. Here we elaborate on the four complementary experimental approaches summarized in the main text, presented in the same order as in the main-text section Future Research Directions.

S1.1 Early Detection through Eye Movement and Retinal Biomarkers

We propose a two-stage, non-invasive screening paradigm. First, smooth pursuit eye movement screening in adolescents using portable eye tracking can detect subtle reductions in pursuit gain and increases in latency, which distinguish high-risk individuals prior to psychosis onset (refs: smooth pursuit endophenotype literature). Second, those flagged would undergo longitudinal pattern electroretinography (ERG) to establish individual retinal dopamine baselines and monitor deviations.

S1.2 Causal Validation in Animal Models

Testing causality requires precise experimental control over retinal dopamine during development. Chemogenetic approaches using DREADDs to selectively modulate retinal dopaminergic amacrine cells can induce controlled dopamine fluctuations while leaving central dopaminergic systems intact. Based on the PerTh framework, animals experiencing retinal dopamine instability during adolescence are predicted to develop aberrant cortical connectivity patterns and enhanced vulnerability to synaptic pruning, mirroring the human phenotype. Following established neurodevelopmental model paradigms, validation would require demonstrating post-pubertal emergence of schizophrenia-relevant phenotypes, including deficits in prepulse inhibition and working memory, together with hypersensitivity to dopaminergic (amphetamine) and glutamatergic (MK-801) pharmacological challenges in early adulthood, thereby linking early retinal dopamine instability to the adult psychosis-like state.[1]

S1.3 Cellular Mechanisms in Patient-Derived Tissue

Human iPSC technology enables direct investigation of retinal pathology using patient-derived cells. By differentiating iPSCs from schizophrenia patients into retinal horizontal cells, which are key modulators of receptive field properties, it is possible to test whether dopamine receptor dysfunction or gap junction abnormalities underlie visual processing deficits. This approach distinguishes between intrinsic cellular pathology and upstream dopamine instability, thereby clarifying therapeutic targets.[2]

S1.4 Therapeutic Implications

If validated, PerTh points to distinct intervention windows across development. In childhood, early detection through retinal biomarkers could identify at-risk individuals before the critical adolescent pruning period, enabling prophylactic stabilization of retinal dopamine function. In adolescence, interventions targeting excessive complement-mediated pruning or enhancing synaptic plasticity could prevent the transition to psychosis. These approaches shift the therapeutic paradigm from

---

[1] The study is currently ongoing within the framework of the Czech Health Research Council (AZV CR) grant No. NW25J-04-00110, titled **Chemogenetically Induced Sensory Noise During Adolescence: A Novel Animal Model of Schizophrenia**.

[2] Parts of the study are currently being conducted as part of the Fulbright-Masaryk Scholarship.

symptom management to primary prevention, targeting the cascade at its sensory origins rather than attempting to repair established cortical damage.
